# Consumption of sweetened, dried cranberries may reduce urinary tract infection incidence in susceptible women – a modified observational study

**DOI:** 10.1186/1475-2891-12-139

**Published:** 2013-10-18

**Authors:** Alexandra E Burleigh, Susan M Benck, Sarah E McAchran, Jess D Reed, Christian G Krueger, Walter J Hopkins

**Affiliations:** 1 Department of Urology, University of Wisconsin School of Medicine and Public Health, 600 Highland Avenue, Madison, WI 53792, USA; 2 Department of Urology, University of Wisconsin Hospital and Clinics, 600 Highland Avenue, Madison, WI 53792, USA; 3 Department of Nutritional Sciences, University of Wisconsin-Madison, 256 Animal Science Bldg, 1675 Observatory Drive, Madison, WI 53706, USA; 4 Department of Urology, University of Wisconsin School of Medicine and Public Health, 1685 Highland Ave, Madison, WI 53705, USA

**Keywords:** Urinary tract infection, Cranberry, Recurrent, *E. coli*, Prevention

## Abstract

**Background:**

Urinary tract infections (UTIs) are one of the most common bacterial infections, and over 50% of women will have a UTI during their lifetimes. Antibiotics are used for prophylaxis of recurrent UTIs but can lead to emergence of drug-resistant bacteria. Therefore, it is reasonable to investigate nutritional strategies for prevention of UTIs. Cranberry juices and supplements have been used for UTI prophylaxis, but with variable efficacy. Because dried cranberries may contain a different spectrum of polyphenolics than juice, consuming berries may or may not be more beneficial than juice in decreasing the incidence of UTIs in susceptible women. The primary objectives of this study were to determine if consumption of sweetened, dried cranberries (SDC) decreases recurrent UTIs and whether this intervention would alter the heterogeneity, virulence factor (VF) profiles, or numbers of intestinal *E. coli*.

**Methods:**

Twenty women with recurrent UTIs were enrolled in the trial and consumed one serving of SDC daily for two weeks. Clinical efficacy was determined by two criteria, a decrease in the six-month UTI rates pre- and post-consumption and increased time until the first UTI since beginning the study. Strain heterogeneity and virulence factor profiles of intestinal *E. coli* isolated from rectal swabs were determined by DNA fingerprinting and muliplex PCR, respectively. The numbers of intestinal *E. coli* eluted from rectal swabs pre- and post-consumption were also quantified.

**Results:**

Over one-half of the patients did not experience a UTI within six months of SDC consumption, and the mean UTI rate per six months decreased significantly. Kaplan-Meier analysis of infection incidence in women consuming SDC compared to patients in a previous control group showed a significant reduction in time until first UTI within six months. The heterogeneity, VF profiles, and prevalence of intestinal *E. coli* strains were not significantly different after cranberry consumption.

**Conclusions:**

Results of this study indicate a beneficial effect from consuming SDC to reduce the number of UTIs in susceptible women. Because there were no changes in the heterogeneity or VF profiles of *E. coli*, additional studies are needed to determine the mechanism of action of SDC for reduction of UTIs.

## Background

Urinary tract infections (UTIs) are one of the most commonly acquired bacterial infections. Over 50% of women will have a UTI during their lifetimes, and approximately 25% of women will have at least one recurrence [1-3]. Recurrent UTIs (RUTI), defined as at least three episodes of UTI in the last 12 months or two episodes in the last six months, can occur in susceptible women and are a significant source of patient morbidity and health care costs [4].

The treatment of RUTIs can vary depending upon the physician and patient. The Infectious Disease Society of America 2010 recommends a five-day course of nitrofurantoin as a first line treatment of acute, uncomplicated cystitis; however, patients may be prescribed other antibiotics as well [5]. Antibiotic prophylaxis can also be initiated in patients with recurrent UTIs. One study showed that 6 to 12 months of antimicrobial prophylaxis was more effective than placebo in reducing the risk of RUTIs in pre- and postmenopausal women [4]. In some cases, patients may self-treat with post-coital antibiotics if RUTIs are related to intercourse [6]. Unfortunately, continuous and sporadic antibiotic use has the potential to select for highly adaptive, multi-drug resistant organisms and can reduce the numbers of beneficial, commensal bacteria in the intestinal flora. Also, long-term use of nitrofurantoin, a common prophylactic antibiotic, has been associated with health risks including pulmonary toxicity, acute and chronic hepatic disease, neuropathy, and anemia [7]. Further, there is no evidence-based recommendation of ideal prophylactic antibiotic, dosage, or duration of treatment for RUTI [8]. Therefore, it is reasonable to consider alternative, readily-available nutritional supplements as prophylactic agents for RUTIs in susceptible women.

Cranberry products have been studied for UTI prevention and treatment. The most common products tested include juices and pills or capsules containing cranberry extracts or powdered fruit, both of which have demonstrated conflicting evidence for prevention of RUTIs [9-11]. One proposed mechanism of action is that one of the primary compounds in cranberries, proanthocyanidins (PACs), inhibit P-fimbriated *E. coli* from adhering to uroepithelial cells [12]. P-fimbriae mediate adherence to uroepithelial cells via the P adhesin gene (*papG*), which contains three unique allelic variants, with the allele III variant being highly associated with acute cystitis [13].

While previous studies have evaluated cranberry juice and other cranberry products for UTI prophylaxis, none have investigated whether consumption of dried cranberries have the potential to decrease the incidence of RUTIs in susceptible women. Dried cranberries contain high concentrations of polyphenols that may be active in interfering with *E. coli* biologic or metabolic processes in the intestine or inhibiting bacterial adherence to vaginal or bladder epithelial cells. In addition, because *E. coli* is the predominant uropathogen isolated from acute and RUTIs in women and it is believed that the infecting bacteria originate from the intestinal flora, the intestinal tract may be an alternate site in which components of the dried cranberries can interact with *E. coli* to reduce infectivity [14,15].

The clinical study presented here investigated the efficacy of consuming sweetened, dried cranberries (SDC) to reduce UTI incidence in women with RUTIs. We observed a reduction in the number of UTIs in the six months following consumption of SDC daily for two weeks. There were no significant changes in the virulence profiles of intestinal *E. coli* during the study period.

## Materials and methods

Twenty women with a history of recurrent UTI, defined as at least three UTIs in the past year (subjects with two UTI’s in the past six months but not three in the past year were also enrolled) provided informed consent at the outpatient clinic of the University of Wisconsin Hospital and Clinics, and were enrolled in the trial approved by the University of Wisconsin Health Sciences Institutional Review Board. The number of subjects was based on achieving a statistical power of at least 80% for each of two parameters, infection-free status at the end of the study, and changes in the *E. coli* strain diversity measure. A six-month infection-free incidence of 60% in 20 UTI-susceptible women consuming SDC compared to a 20% incidence in untreated women would provide 80% power with p = 0.05 using a two-tailed *t*-test. Similarly, analysis of the diversity of *E. coli* strains isolated from 20 subjects pre- and post-consumption would have at least 80% power to detect a 0.66 standard deviation shift in the Kullback Liebler (KL) diversity measure [16] using a two-tailed *t*-test conducted at a significance level of 0.05. The mean age of participants was 37, with a range of 18 to 64, and the mean number of UTIs in the past six months was 2.4 with a range of two to five. Exclusion criteria included immune-compromising diseases, co-morbidities, chronic inflammatory bowel disease, diabetes, or cranberry allergy. Although target enrollment for the study was 20 subjects, data from 3 subjects were removed from the analysis due to one patient with exclusionary criteria realized after enrollment, one patient with sample contamination, and one patient with UTI contracted within one week of study enrollment.

A two-week antibiotic washout period of non-prophylactic antibiotics (i.e. full treatment course for acute UTI) was required before beginning the study. Women who were taking daily prophylactic antibiotics, however, were allowed to participate in the study, provided they met the inclusion criteria. A total of three women who were taking prophylactic antibiotics were enrolled in this study. The women in the study consumed one serving (42 g) of SDC, provided by Ocean Spray Cranberries Inc., each day for two weeks. Rectal swabs were collected at the time of study entry to sample intestinal bacterial flora, and SDC consumption began the following day. Rectal swabs were again collected after two weeks of SDC consumption, and within one day of final SDC consumption.

Clinical efficacy was determined by 1.) Comparison of six-month UTI rates pre- and post-consumption, and 2.) Kaplan-Meier analysis of the time until a patient’s first UTI since beginning the study compared to a previous control group with similar inclusion/exclusion criteria. The six-month UTI rate was based on patient self-reports and documentation of the total number of UTIs in the year prior to the study and six-months since beginning the study. Patients also reported the date of their first UTI since the start of the study.

The presence of *E. coli*, *Enterococcus sp.*, *Klebsiella sp.*, *S. Saprophyticus*, *S. aureus*, and *Proteus sp.* were quantified as colony forming units (CFU) per ml from rectal swab eluates on BBL™ CHROMagar Orientation media (24hr growth at 37°C). *Lactobacillus* was quantified as CFU/ml on deMan Rogosa Sharpe (MRS) *Lactobacillus* agar and grown anaerobically for 72hr at 37°C. In addition, rectal swab eluates were streaked separately onto eosin methylene blue agar to isolate *E. coli*, and the last five colonies at the end of the streak area, including any other morphologically distinct colonies, were stored for further analysis. This method provides ≥97% probability of capturing the predominant strain in the sample [17].

*E. coli* strain heterogeneity was determined by Diversilab™ DNA fingerprinting based on ERIC repetitive PCR [18]. Five *E. coli* strains were isolated from rectal swabs pre- and post-consumption, and the number of unique fingerprints was determined. The KL diversity measure was used to determine changes in intestinal *E. coli* heterogeneity pre- and post- SDC consumption [16].

All isolates were confirmed as *E. coli* by amplification of the glutamate decarboxylase (*gad*) gene in addition to the colorimetric reaction on BBL™ CHROMagar Orientation media [19]. Mulitplex PCR was used to determine phylotype (A, B1, B2, or D) and the presence of six pathogenicity islands (PAIs) and 15 VFs related to iron-uptake, adhesion and toxicity [20-22]. The PAIs tested were from *E. coli* J96 (II_J96_), *E. coli* CFT073 (I_CFT_, and II_CFT_) and *E. coli* 536 (I_536_, II_536_, and IV_536_) [23]. The individual siderophore genes tested included *fyuA*, *iroN*_*E.coli*_, and *iutA* . The adhesion genes tested were *sfa*, *papG allele II/III*, *sfaS*, and *iha*. The toxins included *hlyA*, *cnf1*, *pic*, *tsh*, *sat*, and *tosA*, and other genes tested were *K1* and iss.

Two separate statistical analyses were used to evaluate clinical efficacy. A two-tailed paired *t*-test and mean differences with standard errors (SE) was used to determine significant differences in the six-month UTI rates pre- and post-consumption. A log-rank test was used to compare the time until first UTI between the current study group and two placebo groups from previous clinical trials. The two placebo groups from the previous trials were pooled (p = 0.83).

Significant differences in the numbers (CFU/ml) of bacteria present pre- and post-consumption were determined using the Wilcoxon matched pairs signed rank test using log-transformed values to calculate geometric means with 95% confidence intervals (CI), and the presence of VFs was expressed as percentages.

## Results

### Clinical efficacy of SDC consumption in UTI-susceptible women

**Figure 1:**
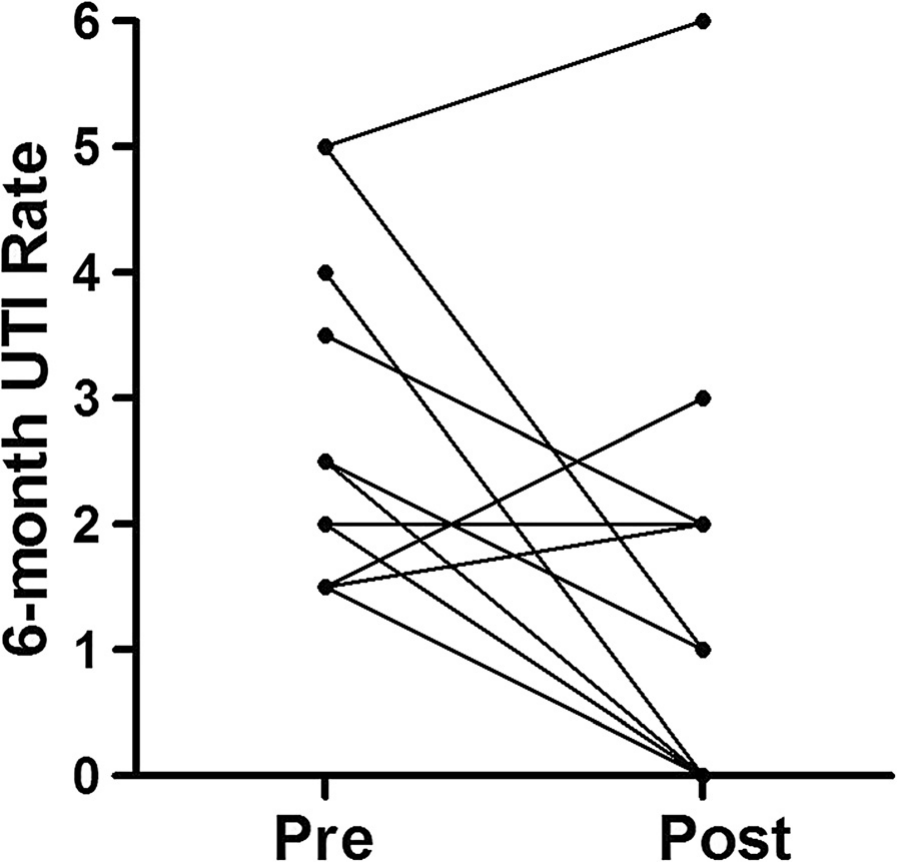
**Six-month UTI rates Pre- and Post- sweetened dried cranberry consumption.** In 17 patients, the mean pre- and post- SDC consumption UTI rates were 2.4 and 1.1, respectively (p = 0.004). The pre-consumption UTI rate is based on one half of the total number of UTIs in the past year or the total UTIs in six months prior to entering the study. Post-consumption six-month UTI rate is based on the total number of UTIs reported six months since beginning the study.

There was a significant reduction in the incidence of UTIs and time until first UTI recurrence in women consuming SDC, as determined by two separate statistical analyses. First, the six-month UTI rate was compared pre- and post-consumption. Nine of 17 (53%) patients reported no UTIs within six months since beginning the study, and the mean UTI rate per six months decreased significantly from 2.4 to 1.1 (Figure 1, p = 0.004, mean difference = 1.26 ± 0.38SE). Since three patients were taking daily antibiotic prophylaxis which may affect the results, the clinical efficacy was determined with and without these patients. When these women were excluded from analysis, the six-month UTI rate also significantly decreased (p = 0.010, mean difference = 1.18 ± 0.39SE).

**Figure 2:**
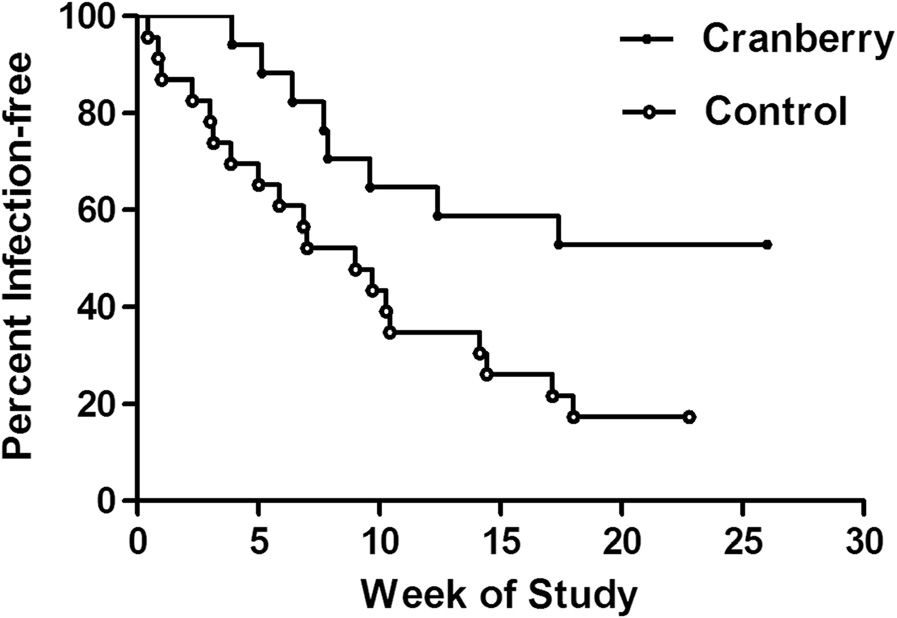
**Increased time until first UTI following two-week consumption of sweetened dried cranberries.** Seventeen patients consumed cranberries for the first two weeks of the study and were followed for a total of six months. Twenty-four control patients were given three doses of a placebo at one-month intervals in a previous vaccine trial. Kaplan-Meier analysis of patients remaining infection-free following SDC consumption compared to a previous control had p = 0.023.

Second, Kaplan-Meier analysis compared the incidence of UTIs in study patients to patients receiving a placebo in two previous UTI vaccine trials [24]. Women in the current and previous trials had equivalent UTI histories. This analysis showed a significant reduction (p = 0.023) in the acquisition of UTIs within six months for patients consuming SDC (Figure 2). Fifty-three percent of women consuming SDC were uninfected compared to 20 percent of women receiving placebo who were uninfected six-months since beginning the study. When the three women who were taking prophylactic antibiotics were removed from the Kaplan-Meier analysis, the results were more statistically significant since the majority of women taking prophylactic antibiotics still acquired infections (p = 0.018).

### Characterization of intestinal bacterial flora pre- and post- SDC consumption

**Figure 3:**
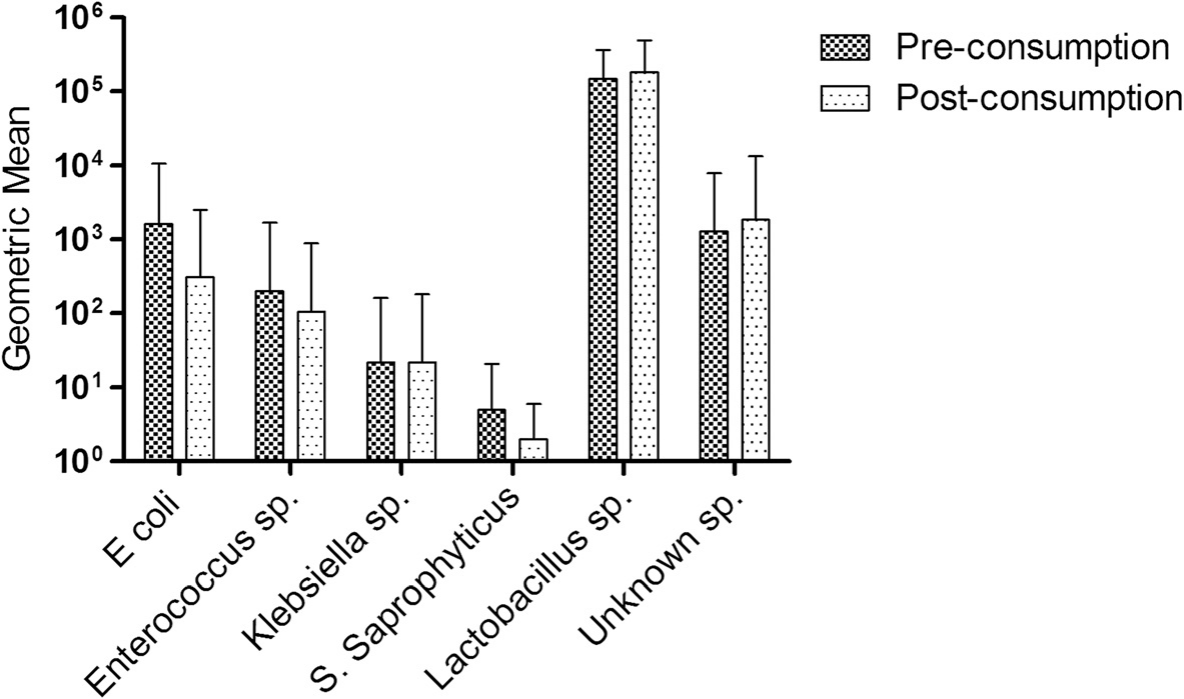
**Bacterial species present in rectal swab eluates pre- and post SDC consumption.** Types of bacteria isolated from rectal swab eluates at study entry and two weeks after consuming sweetened, dried cranberries. Bacterial cell counts are presented as geometric means (CFU/ml) with 95% CI.

Based on the positive clinical implications of SDC consumption, further microbiological analysis determined whether the intestinal flora was altered by SDC consumption. There was a significant decrease (p = 0.040) in the geometric mean of CFU/ml of *E. coli* isolated from rectal swab eluates pre- and post-consumption (Figure 3). There were no significant increases or decreases in *Enterococcus sp.* (p = 0.315), *Klebsiella sp.* (p = 0.734), *S. Saprophyticus* (p = 0.875), *Lactobacillus sp.* (p = 0.600) or Unknown sp. (p = 0.508). *S. aureus* and *Proteus sp.* were not isolated from any rectal swab eluates pre- or post-consumption.

### Heterogeneity of intestinal E. coli strains

To determine changes in *E. coli* heterogeneity pre- and post-consumption within each patient, Diversilab™ DNA-fingerprint banding patterns for each rectal *E. coli* strain were compared using KL clustering, and strains that were ≥95% similar were considered identical and those <95% similar were considered unique. The overall diversity measure was calculated for each patient, and those with both pre- and post-consumption rectal *E. coli* present were analysed (n = 11). Based on the number of unique *E. coli* strains isolated, the KL diversity measure was calculated to determine any changes in heterogeneity after SDC consumption. Seven of eleven patients (64%) had no change in the diversity measure, 2/11 (18%) had increased heterogeneity (A16 and A18), and 2/11 (18%) had decreased heterogeneity post-consumption (A7, A11). The overall mean diversity measure was 0.0080, which indicates that there was no change in the heterogeneity of *E. coli* after consumption of SDC (Table 1). Also, it is important to note that 8/11 (72%) of the patients had at least one dominant *E. coli* strain that was present before and after SDC consumption.

**Table 1:** **Diversity measures of rectal *E. coli *strains pre- and post-consumption of SDC**

**Diversity change**^**1**^	**Pre**		**Post**		**Diversity measure**^**3**^
	**Types**^**2**^	**Diversity**	**Types**	**Diversity**	
None					
A1	5/0	0	5/0	0	0
A5	5/0	0	5/0	0	0
A8	5/0	0	5/0	0	0
A9	5/0	0	5/0	0	0
A13	5/0	0	5/0	0	0
A14	4/1	-0.2170	4/1	-0.2170	0
A19	5/0	0	5/0	0	0
Increase					
A16	4/1	-0.2170	3/1/1	-0.4130	-0.196
A18	3/2	-0.2920	2/2/1	-0.4580	-0.166
Decrease					
A7	2/2/1	-0.4580	4/1	-0.2170	0.241
A11	4/1	-0.2170	5/0	0	0.217
**Total mean**		**-0.1168**		**-0.1088**	**0.0080**

^1^Diversity change indicates whether the number of unique strains increased, decreased, or stayed the same after SDC consumption. Patient number 1 is identified as A1, patient number 5 as A5, etc.

^2^Five *E. coli* strains were isolated from rectal swabs pre- and post-consumption, and the number of unique fingerprints was determined using Diversilab™ DNA-fingerprinting. Strains were compared using KL clustering, and strains that were ≥95% similar were considered identical and those <95% similar were considered unique. A type designated as 5/0 means that all 5 strains were ≥95% similar and there were no unique strains.

^3^When the DNA fingerprints of all five rectal *E. coli* isolates were the same, the diversity measure was zero. As the number of unique fingerprints increased, the diversity measure becomes an increasingly negative number.

The *E. coli* strains were further characterized by phylotype and the prevalence of 15 VFs and 6 PAIs for each unique strain. The VFs tested were those involved in toxin production, adhesion, and iron regulation. Of the phylotypes, VFs, and PAIs tested, only two of the phylotypes, one iron-regulation gene (*iutA),* and one PAI (I_CFT_) changed by more than ten percent after SDC consumption. The least pathogenic phylotypes, A and B1, increased by 8% and decreased by 14%, respectively, and the more pathogenic phylotypes, B2 and D, decreased by 8% and increased by 11%, respectively. The proportion of *E. coli* strains with *iutA* increased from 6/21 (29%) to 9/21 (45%) after SDC consumption and the proportion of I_CFT_ decreased from 10/21 (48%) to 7/21 (35%). All of the other VFs and PAIs tested did not change by more than ten percent.

## Discussion

Here we report the results of a modified observational clinical trial of two-week SDC consumption for the prevention of UTIs in susceptible women. Using two different methods to determine efficacy, consumption of SDC was shown to be beneficial in reducing the occurrence of UTIs in susceptible women. The significant decrease in six-month UTI rates per individual patient and the comparison of UTI recurrences to a previous control group of women with equivalent UTI histories provide evidence of the potential prophylactic effect of SDC.

Based on the significant decrease in UTI incidence, potential mechanisms of SDC action were investigated. We examined whether the reduction in UTIs could be accounted for by significant changes in the numbers or prevalence of intestinal *E. coli* or other bacterial species following SDC. *E. coli* was the only bacteria that showed a significant decrease in overall numbers over the course of the study. It is possible that polyphenols from SDC blocked adherence of *E. coli* to the intestinal surface and thus decreased their overall numbers, rather than exerting a bactericidal effect. The lack of SDC toxicity is also supported by the observation of no significant changes in the numbers of the other intestinal bacteria surveyed. It is thus likely that SDC are not affecting the growth of commensal bacteria, which is contrary to what would occur if patients were taking prophylactic antibiotics. The lower numbers of intestinal *E. coli,* combined with the lack of interference with other types of bacteria, may provide a potential benefit since it produces no adverse effects and results in decreased likelihood of UTI.

Although there were no changes in the heterogeneity of intestinal *E. coli* strains after SDC consumption, we questioned whether there were differences in the intestinal *E. coli* phylotypes or virulence gene profiles after SDC consumption. The proportion of *E. coli* with A and D phylotypes was higher after SDC consumption than *E. coli* with B1 and B2 phylotypes. Phylotypes A and B1 are considered to be less uropathogenic than D and B2. Thus, the increased presence of the A phylotype and decreased presence of the B2 phylotype could be beneficial; however, this does not explain the increased and decreased presence of *E. coli* with D and B1 phylotypes, respectively. Although there were changes in the phylotypes of *E. coli* pre- and post-consumption, there was little change in the proportions of strains carrying VF genes associated with UTI. The prevalence of only the *iutA* and PAI I_CFT_ VF genes in strains differed by more than ten percent after SDC consumption. Overall, the heterogeneity of *E. coli* and the presence of VFs did not change as a result of SDC consumption, which suggests that SDC do not directly influence the underlying make-up of *E. coli* in the intestinal flora.

Because the significant reduction in the number of UTIs following SDC consumption is most likely not related to reducing the prevalence or heterogeneity of intestinal uropathogenic *E. coli* or other potential uropathogens, alternative mechanisms whereby cranberry phytochemicals could directly influence bacterial phenotypes should be considered. One intriguing possibility is that some components of cranberries may be able to modulate expression of *E. coli* VF genes and reduce infectivity and pathogenicity. Support for this concept comes from several studies. Trans-cinnamaldehyde was shown to decrease *E. coli* attachment to uroepithelial cells and to reduce cellular invasion in vitro by downregulating expression of the *fimH* gene coding for the adhesin of type 1 pili [25]. Cinnamaldehyde and some of its derivatives were also shown to reduce the virulence of *Vibrio* species by decreasing the quorum sensing response [26]. It has also been reported that compounds in crushed cranberries or cranberry proanthocyanidins inhibited expression of the *fliC* flagella structural gene in the CFT073 UPEC strain, with subsequent loss of motility [27]. Resveratrol, which is present in cranberries, decreased the pathogenicity of *Proteus mirabilis* by inhibiting swarming as well as reduced hemolysin production and urothelial cell invasion [28]. It is possible that these, or other compounds found in cranberries could similarly decrease the virulence of intestinal *E. coli* by gene modulation that reduces motility as well as uroepithelial cell adherence and invasion, thereby reducing uropathogenic *E. coli* persistence and infectivity in the vagina and bladder.

Another potential mechanism to consider for the observed clinical benefits of SDC consumption is that cranberry phytochemicals may boost mucosal immunity to uropathogens in the gastrointestinal tract. A recent study has shown that cranberry proanthocyanidins can be used as an adjunct to increase intestinal *sIgA* levels in mice receiving enteral nutrition [29]. A clinical investigation found that consuming a cranberry beverage for ten weeks resulted in a five-fold increase in proliferation of T cells ex vivo, a 30% increase in NK cell proliferation, and a 20% reduction in IL-17 secretion, as well as a significant reduction in cold and flu symptoms compared to placebo [30]. Further studies on the immune enhancing effects of SDC in vivo could define which components of the intestinal immune system are targeted by cranberry phytochemicals.

Although SDC consumption did not prevent UTIs in every patient, none of the patients reported any adverse effects. Therefore, it may be suggested that SDC be used as a prophylactic supplement for patients with recurrent UTIs, since the potential benefits greatly outweigh the risks. A larger, randomized, placebo-controlled study is needed to confirm that daily consumption of SDC is beneficial in the reduction of recurrent UTIs.

One of the limitations of the efficacy assessment is the comparison of patients consuming SDC in this study to a control group from a previous study. We are confident, however, that this comparison between groups is applicable and informative even though it is not the most appropriate comparison for rigorous statistical analysis. Because the women in the current trial and the previous placebo-controlled study had equivalent UTI histories and similar demographics, we would expect women in both groups to acquire UTIs at the same rate over a six-month period without treatment interventions. Randomized, placebo-controlled studies are necessary to further determine the efficacy of SDC consumption.

## Conclusions

The results of this study indicate a potential beneficial effect of consuming SDC in reducing the number of recurrent UTIs in susceptible women; however, the results shown here are only preliminary and further studies are necessary. Daily SDC consumption is an inexpensive and readily available supplement to a woman’s diet and may provide potential prophylactic effects. Despite the observed clinical efficacy of SDC consumption, however, the underlying mechanism of SDC is still unclear. Two-week consumption of SDC reduces the overall numbers of *E. coli* in the intestines but does not change the *E. coli* strain heterogeneity or the presence of other bacterial types. Future studies are needed to determine the underlying mechanism of SDC for reduction of UTIs in susceptible women.

## Competing interests

The authors declare they have no competing interests.

## Author contributions

AB recruited patients for the study, obtained informed consent from subjects, conducted laboratory assays, analyzed data, and drafted the manuscript. SB was the clinic nurse for the study and interacted with study subjects. SM assisted with the development of the study design and recruited patients for the study from her clinical practice. JR and CK helped with the development of the study design and provided expertise on cranberry phytochemicals. WH was the principal investigator and provided oversight for the study. WH developed the study design and methods, and assisted with drafting the manuscript. All authors read and approved the final manuscript.
